# Review of The Palgrave Handbook of Applied Linguistics Research Methodology, by Aek, Phakiti; Peter De Costa; Luke, Plonsky; & Sue, Starfield

**DOI:** 10.1186/s40862-020-00095-x

**Published:** 2020-08-10

**Authors:** Kioumars Razavipour

**Affiliations:** Shahid Charmran University of Ahvaz, , Ahvaz, Iran

**Keywords:** Applied linguistics, Research methodology, Data collection methods, Data analysis methods

## Introduction

From an early limited conceptualization as the application of linguistic knowledge to language teaching (Cook, 2015), current Applied Linguistics (AL) is a vibrant interdisciplinary field that considers within its domain any social problem in which language is implicated (Brumfit 1995, cited in Myers, 2005). Given that it is difficult to imagine a sphere of social life in which language is not implicated (Myers, 2005), demarcating the borders of AL research is difficult to articulate (Chapelle, 2013; Cook, 2015; Kaplan, 2010). This continuous diversification in scope requires that “researchers ask and address different research problems, employing a variety of philosophies and methodologies.” (Phakiti, et al. 2018, p. 6). The expansion in both the substantive domain and in the array of data sources and analytical tools that are used in AL research demands that the repertoire of research methods in AL undergo continual updates and critique. The handbook edited by Phakiti et al. is a major step in this direction.

## Review

*The Palgrave Handbook of Applied Linguistics Research Methodology,* edited by Phakiti, De Costa, Plonsky and Starfield, was published by Palgrave. The editors are among the established methodologists of the field and have widely published on various methodological issues in AL research.

Comprising of 41 chapters, the handbook is organized in four parts around five stages of empirical research including thinking, designing, collecting, analyzing, and disseminating (p. 13). The first part, dealing with *research approaches and methodology,* comprises of ten *chapters*. In the first chapter, the editors justify the need for the handbook by making reference to the recent heightened awareness of methodological issues in AL research and the need for methodological reform in the field. They then provide a taxonomy of various approaches to research in AL and put forward a research cycle model that informs the organization of the handbook. This cycle consists of the five stages of thinking, designing, collecting, analyzing and disseminating.

In chapter two, Young poses very fundamental challenges to our thinking through highlighting the role of habits of mind in shaping researchers’ thought-styles and their epistemologies. Drawing on Fleck’s sociology of knowledge and his notions of rhetoric, epistemology, and incommensurability, Young argues that differences in research methods arise from differences in rhetoric and epistemology. This echoes critical calls in AL (Corson, 1997; McNamara, 2015; Pennycook, 2018) inviting scholars to defamiliarize the familiar and question the taken-for-granted in AL research. The importance and limitations of quantitative research are discussed in the third chapter and alignment of analysis to the research problem is emphasized. Contrasting the quantitative method as reducing data to numbers with qualitative method as “reduction of data to words” (p. 80), the next chapter focuses on the history, philosophy and application of qualitative research. The rather old dichotomy of the numerical and the verbal is often followed by a marriage of the two in mixed methodology, discussed in chapter five, which elaborates on the rationale, the advantages, different designs, and limitations of mixed research methodology. Literature review is the common denominator of all research methods. Making a distinction between the twin secondary research approaches of traditional literature review and research synthesis and discussing the merits of each, chapter six makes a case for the latter to be treated “as a method in its own right” (p. 124). In Chapter seven, Abbual traces the evolution of replication research, elaborates on “exact, approximate, and conceptual” types of replication (p. 145), argues convincingly that replication is not “the poor cousin of original research” (p. 146), and problematizes the possibility and desirability of replicating qualitative research. As in other spheres of life, ethical conduct in research is of prime importance. In chapter eight, Sterling and De Costa broaden the scope of research ethicality to encompass data collection and representation, the communication of findings, and the wider issues of social responsibility in conducting research. Emphasizing the value and utility dimensions of research, they maintain that “research should be conducted only if it is deemed to be useful or worthwhile” (p. 165), implying that research which serves only the career advancement of the researcher is of dubious ethicality. Chapter nine, written specifically for the doctoral students, walks the reader through the major stages of writing a research proposal. In the final chapter of this part, Samraj reviews findings from genre analysis on both macro and micro structure of the research article genre and draws implications and guidelines for writing and publishing research papers.

Part two of the volumes is almost entirely about the collecting stage of the research cycle, which underlies the organization of the handbook. In chapter 11, Prior makes a distinction between interview as a research instrument that “aligns with the transition or conduit metaphor model of communication” (p. 229) and the constructivist approach that views interview as social practice. He then discusses interview formats on a continuum of standardized to less structured, conversational interviews. Relatedly, observations and field notes are dealt with in the next chapter, where Copland elaborates on the essential processes of making field notes. Chapter 13 is of particular relevance to doing research in the Covid-19 era. In this chapter, Dewaele lays out challenges associated with online questionnaires such as heterogeneous samples, self-selection bias, and validity. In Chapter 14, Grey and Tagarelli address the pros and cons of various cognitive measures and tasks such as eye tracking and response time. A perennial challenge in AL research is the elicitation of the right data from the participants. This is addressed in the next chapter, where Gass addresses the strengths and limitations of elicited imitation and grammaticality judgement tasks in tapping into explicit, implicit, and metalinguistic knowledge. Relatedly, in chapter 16, Bowels evaluates the validity and usefulness of think-aloud and stimulated recall as two commonly used elicitation tools to probe into the process of language learning. Paquot then gives an overview of the potential uses of corpus-driven research in language pedagogy “by informing decisions about what to teach and when to teach it” (p. 359). Hafner’s chapter on the challenges and opportunities that digital discourses present to applied linguistics closes this part. In specific, he addresses the ethicality, collection and analysis of digital discourse data for AL research.

Part three of the volume contains ten chapters on data analysis, the fourth stage of the research cycle. In Chapter 19, Norouzian and Plonsky address theoretical and statistical issues related to correlation and multiple regressions, as used in “analyses of associations (p. 396) and quantitative instrument validation. As AL scholars embark on addressing more complicated social problems, so must their analytical tools become more sophisticated. Contributing to this end, in the next couple of chapters, Phakiti walks the reader through exploratory factor analysis (EFA) and confirmatory factor analysis (CFA). In AL research, comparing group means is as old as the field but it is often done inconsistently and in violation of standards of best statistical analysis practice (p. 501). Amoros’s chapter on the family of Analyses of Variance (ANOVA) provides both a conceptual rationale and practical tips for conducting and interpreting means-based comparisons. Another widespread challenge in AL research is that the data we work with is often non-traditional (p. 524) in that they do not meet the assumptions of parametric statistics and therefore require alternative methods of analysis. Egbert and LaFlair explain how non-nominal data, data coming from small samples, and non-normal data can be properly analyzed using non-parametric statistics, bootstrapping, and permutation tests. The quality of quantitative data directly bears on the validity of findings (Plonsky & Derrick, 2016). In chapter 24, Grabowsky and Oh discuss the reliability of quantitative data as a crucial research transparency requirement and provide guidelines for investigating and minimizing external and internal sources of unreliability. In the next chapter, Crossley and Kyle go beyond conventional discourse and corpus analyses and explain how big data can be analyzed using machine learning and natural language processing technology. Story telling is perhaps the oldest genre of human discourse. Building on this premise, In Chapter 26, Benson elaborates on the epistemological grounds that have contributed to the return of narrative inquiry as a popular research method. In particular, he points to the fact that many AL scholars “have become weary of variables and the quantification of the positivistic approach” (p. 597). He then distinguishes between discourse and content approaches to narratives and focuses on narrative analysis in the remainder of the chapter. The next chapter addresses qualitative analysis of interaction as discursive practice between people with particular focus on interaction in language teaching contexts. The final chapter in this part broadens the scope of data to include, in addition to text, other semiotic resources such as pictures, images, and the physical environment in researching social issues of concern to applied linguists.

In the final part of the handbook, research methods in ten selected AL areas are addressed. This part opens with quantitative research methods in instructed second language acquisition (SLA), where Loewen discusses the construct validity of measured variables in SLA. This is followed by Bahtia’s chapter, which is a state-of-the-art treatment of bilingualism and multilingualism. In chapter 31, after giving an overview of major research areas, Larner discusses ethicality and data collection challenges in forensic linguistics. With the global spread of English, how to conduct research into the characteristics and norms of World Englishes is a major challenge. De Costa, Maloney, and Crowther take up this issue addressing corpus-based, ethnographic, acoustic and survey-based approaches to doing research on World Englishes. In chapter 33, various orientations to research in heritage and community languages are discussed by Bhalla and Wiley. This is followed by Angelelli’s chapter, which begins with tracing the evolution of the field of translation and interpreting. She then discusses the triple challenges in the field namely, the gulf between theory and practice, the status and professionalization of translators, and translation pedagogy. Another key issue that is covered in this part is identity, which is, according to Darvin, “inextricably intertwined with language” (p. 777). Darvin addresses four major research methods used in identity research: case study, narrative inquiry, conversation analysis, and critical discourse analysis. Related to the question of identity is how language and discourse co-occur with kinesics. In chapter 36, after giving an overview of research on gestures in AL, Stam and Buescher discuss issues of research method, design, and analysis in researching gestures. Given the new surge of interest in new materialism (Pennycook, 2018), this line of inquiry is likely to witness increasing interest in the coming decades. In the next chapter, Johnson and Stephens trace the theoretical and methodological evolution of research in language policy and planning, and emphasize the need to take account of discursive practices and social impact. In chapter 38, Youn first provides an overview of research on learning, teaching, and assessment of L2 pragmatics and then discusses data collection procedures, especially Discourse Completion Tests and role plays. Throughout the chapter, Youn emphasizes the constant tension between construct representation and practicality issues in L2 pragmatics studies and emphasizes that the choice of data should not be driven by “convenience or convention” (p.835) but should be informed by the nature of research problem under consideration. A note on the pedagogy and assessment of pragmatics in World Englishes would have made the chapter even richer. In chapter 39, after giving an overview of language testing, Ginther and McIntosh discuss cores issues of validity, communicative competence models, proficiency scales developed by American Council on the Teaching of Foreign Languages (ACTFL) and by Common European Framework of Reference (CEFR), classical test theory, and research methods. At the conclusion, they concentrate on three perennial issues of consequential validity, assessment literacy, and World Englishes. In Chapter 40, which is about moving from a dominant concern with language in the social to language on the physical environment, Malinowski emphasizes methodological diversity and uncertainty in researching linguistic landscape (LL) of urban areas**.** He then elaborates on the evolution of LL research methods from an early positivist approach to ethnography and to the more recent symbiotic, mixed-methods approaches. Finally, the handbook closes by a discussion of two major approaches to researching academic literacy namely, academic literacy as a cognitive process and academic literacies as socialized identity construction within discourses and communities of practice.

### Strengths of the handbook

As the above summary indicates, this volume has a number of advantages. First, the volume treats issues in AL research at various levels of abstraction, ranging from the very abstract questions of philosophy of science and methodology to more concrete issues such as how gesture data can be properly analyzed. The second advantage is that the chapters have been written in an accessible language. As a field of knowledge matures, the established members of its discourse community may unconsciously come to take for granted assumptions, knowledge and beliefs that are not necessarily shared by those who are new to the discourse community. An outstanding feature of this volume is that in its treatment of each research method, data collection, or data source, it does not assume much background on the part of the reader. Managing to do so in the scope of a chapter is no easy undertaking, which the editors and the authors have successfully accomplished. Thirdly, the volume is at same time both broad and specific. The editors rightly note in the introductory chapter, that existing research methods textbooks are either about the general nature of research (e.g., Mackey & Gass, 2015) or focus on a specific research method (e.g., Duff, 2008, on case study). The fact that this handbook combines the features of both types of the noted research textbooks adds to the strengths of the handbook. Therefore, the volume can be relied upon as a self-contained, autonomous guide to various stages of the research cycle from inception to publication. The fourth strength of the handbook is its comprehensiveness. Though absolute comprehensiveness is not achievable given the scope of AL, the handbook is relatively comprehensive in its treatment of various research methods, data collection instruments, data analyses and research methods in diverse substantive areas of AL. Another advantage of the volume is that at the end of each chapter, a list of key resources is provided for the readers who are interested to pursue the theme of the chapter further. Last but not least, after providing the theoretical background to the topic at hand, in most chapters the reader is walked through an actual or simulated example so that the reader can see how each research or analysis method is used in practice.

### Limitations

On a critical note, the handbook could have been even more comprehensive had the two areas of language teacher education and the validity of research instruments received more explicit coverage, because consistency of data elicitation instruments, covered in the handbook, is only one of the requirements of proper research instrumentation, as Purpura, Brown, and Schoonen (2015) maintain. Another minor point is that, though chapters are not meant to be organized on a continuum of difficulty, in some cases the ordering of chapters could be otherwise. For instance, the chapter on Analysis of Variance could have come before the two chapters on Exploratory Factor Analysis and Confirmatory Factor Analysis, which are more sophisticated statistical issues than ANOVA.

Overall, to me, the editors and the contributors of the handbook have accomplished an outstanding job. The handbook brings together a considerable portion of the latest scholarship and wisdom of the field of Applied Linguistics. This volume is going to be an asset in the personal library of anyone involved in Applied Linguistics research for the coming years.

## Data Availability

Data sharing not applicable to this article as no datasets were generated or analyzed during the current study.
